# Disturbance of osteonal bone remodeling and high tensile stresses on the lateral cortex in atypical femoral fracture after long-term treatment with Risedronate and Alfacalcidol for osteoporosis

**DOI:** 10.1016/j.bonr.2021.101091

**Published:** 2021-05-07

**Authors:** Fumitaka Hirano, Kayoko Furukawa Okuma, Yukichi Zenke, Kunitaka Menuki, Hideo Ohnishi, Fumio Fukuda, Akinori Sakai, Noriaki Yamamoto, Taketoshi Shimakura, Hiroshige Sano, Yuta Tokunaga, Hideaki E. Takahashi

**Affiliations:** a University of Occupational and Environmental Health, School of Medicine Department of Orthopaedic Surgery, 1-1 Iseigaoka, Yahatanishi-ku, Kitakyushu-shi, Fukuoka 807-8555, Japan; b Moji Medical Center, 3-1 Higashiminatomachi Moji-ku, Kitakyushu-shi, Fukuoka 801-8502, Japan; c Kitakyushu General Hospital, 1-1 Higashijonocho Kokurakita-ku, Kitakyushu-shi, Fukuoka 802-8517, Japan; d Niigata Rehabilitation Hospital, 761 Kizaki, Kita-ku, Niigata-shi, Niigata 950-3304, Japan; e Niigata Bone Science Institute, 761 Kizaki, Kita-ku, Niigata-shi, Niigata 950-3304, Japan; f Niigata University Graduate School of Medical and Dental Sciences Division of Orthopaedic Surgery, 757 Asahimachidoriichibancho, Chuo-ku, Niigata-shi, Niigata 951-8510, Japan; g Niigata University of Health and Welfare Graduate School, 1398 Shimami-cho, Kita-ku, Niigata-shi, Niigata 950-3198, Japan

**Keywords:** ASBMR, American Society for Bone and Mineral Research, AFF, atypical femoral fracture, BMD, bone mineral density, YAM, young adult mean, FEA, finite element analysis, LIPUS, low-intensity pulsed ultrasonography, TKA, total knee arthroplasty, Atypical femoral fracture, Osteoporosis, Bone histomorphometry, Finite element analysis

## Abstract

An 83 year-old Japanese woman complained of left lateral thigh pain following a low-energy fall 4 months prior to admission. She had been treated for osteoporosis with Risedronate and Alfacalcidol for the previous five years. She was diagnosed with an atypical femoral fracture (AFF) according to the American Society for Bone and Mineral Research (ASBMR) Task Force revised criteria. Radiographs revealed cortical thickening and a transverse radiolucent fracture line in the lateral cortex of the shaft. MRI showed a high intensity signal on the T2WI image 1 cm long in the lateral cortex. The patient had normal levels of bone resorption and formation biomarkers except for low 25(OH) Vitamin D. Double fluorescent labeling was done preoperatively.

Due to significant bowing, a corrective osteotomy and intramedullary nailing were performed, and the resected bone wedge was analyzed by bone histomorphometry. Three ground sections of the lateral cortex at the fracture site showed many and large pores, with or without tetracycline labeling. Histomorphometric assessment was done on intracortical pores, classified by a novel criteria, only to assess size of the pores to know prolonged osteoclastic activity and its characteristics of inner surfaces to assess whether bone formation has been occurring or not in labeling period in remodeling cycle, and coalition of multi-pores. Increased size with widespread variation of pores suggested prolonged osteoclastic activity in the reversal/resorptive phase. Bone labeling showed lamellar bone on the endocortical surface.

We hypothesize that the case had developed from a regional disturbance of osteonal remodeling in the lateral cortex, in which accumulated microcracks might have initiated a resorption process resulting in resorption cavities, i.e., pores, which became larger due to prolonged activity of secondary osteoclasts. Various sized pores could form lamellar bone, still forming at the time of biopsy, some had formed lamellar bone, but stopped to form before labeling and not to start to form at all, probably due to incomplete coupling. Endocortical lamellar bone might had started to resorbed to smooth off endocortical surface, followed by formation of lamellar bone. The endocortical bone formation was assessed and its formation period is about 2.7 years.

A finite element analysis using preoperative CT data revealed high tensile stresses on the lateral aspect of the femur. Histomorphometric results suggest that there might be more pores in the tensile area than the compressive area. These findings may subsequently connect accumulation of microcracks, an increase of size and number of pores and coalition and subsequent fracture in the lateral cortex.

## Highlights

• The lateral cortex of the fracture site of atypical femoral fracture was assessed by bone histomorphometry and FEA.
• Many enlarged pores may suggest a prolonged resorptive phase, resulting in excessive resorption by secondary osteoclasts.
• There is large variation in size of pores, which is much more than that of osteons, normally observed.
• Pores were classified as types with/without label, and with/without parallel lamellae to inner surface of the pores.
• More pores in size and number were observed in the lateral cortex under tensile force than compressive force by FEA.

## 1 Introduction

Although the etiology of an atypical femoral fracture (AFF) was considered to be associated with the adverse effects of over suppression of bone turnover by the long-term use of bisphosphonates (Odvina et al., 2005; Neviaser et al., 2008; Adler et al., 2016; Mahjoub et al., 2016), which can lead to a stress or insufficiency fracture. But AFF have also been reported in unexposed patients, those receiving denosumab for osteoporosis and metastatic bone disease (Takahashi et al., 2019). Lateral femoral bowing and varus hip geometry, which increase loading forces on the lateral femoral cortex, may increase AFF risk (Oh et al., 2014, Oh et al., 2017; Saita et al., 2014; Haider et al., 2019).Altered bone material properties associated with BP therapy may predispose to AFFs by permitting initiation and increasing propagation of microcracks (Shane et al., 2010, Shane et al., 2014; Starr et al., 2018).

Little histomorphometric findings at the fracture site of AFFs has been reported. Incomplete fractures showed a fracture gap containing an amorphous, non-mineralized material, callus, cartilage matrix and fragments of lamellar bone, probably broken off from the fracture surface in the gap. Adjacent to the fracture line, many resorption cavities and channels are observed and tended to be oriented perpendicular to the fracture plane, but some channels run transversely (Schilcher et al., 2014; Oh et al., 2020). Osteoclasts were frequently found close to the fracture line in the resorption cavities and less frequently further away. About a quarter of these osteoclasts were giant cells with pyknotic nuclei that were adjacent to superficial resorption cavities (Weinstein et al., 2009). There were also a report of that after labeled bone biopsied specimen close to the fracture line (Jamal et al., 2011).

It has been recently well understood the sequence of events in osteonal remodeling of the cortical bone of the metabolically normal iliac crest (Andreasen et al., 2018). The bone close to the fracture gap in this case showed signs of bone remodeling. Because of limited amounts of the specimen from the fracture site, histomorphometric assessment was done on intracortical pores, classified by a novel criteria, only to assess the size of the pores to know cumulative osteoclastic activity and its characteristics of inner surfaces of the pores to assess whether bone formation has occurred, had occurred, or had not occurred after a reversal-resorptive phase in a remodeling cycle.

In spite of the fact that the microcracks were accumulated in the femur it is still unclear how it could develop into the fracture gap. The histomorphometric results might explain how the fracture line could have developed and when the endocortical surface would have started form lamellar bone. The results were corresponded with the finding of a CT-based finite element analysis (FEA), revealed tensile stresses on the lateral femoral cortex, where many large pores are observed.

## 2 Materials and methods

### 2.1 Patient background and surgery

**Fig. 1:**
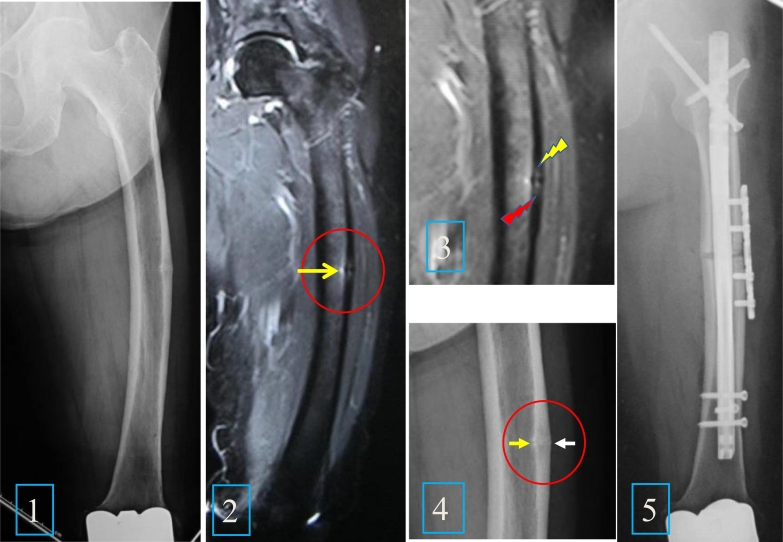
Preoperative X-ray, MRI and post-operative X-ray. 1-1. Preoperative X-ray of the left femur, with lateral bowing and hypertrophy of the lateral cortex of both periosteal and endocortical surfaces of the midshaft. 1-2. Preoperative MRI of the left femur, showing high intensity area in the bone marrow adjacent to the mid-lateral cortex (yellow arrow). 1-3. Preoperative MRI of the left femur, showing high intensity area in the intracortical region of the mid-lateral cortex (upper end: yellow arrow; lower end: red arrow). 1-4. Preoperative X-ray of the left femur. An enlarged image of the midshaft, indicating the hypertrophy of both periosteal (white arrow) and endocortical (yellow arrow) surfaces. 1-5. Postoperative X-ray of the left femur: lateral bowing was corrected and fixed with intramedullary rodding and plate fixation. (For interpretation of the references to colour in this figure legend, the reader is referred to the web version of this article.)

**Fig. 2:**
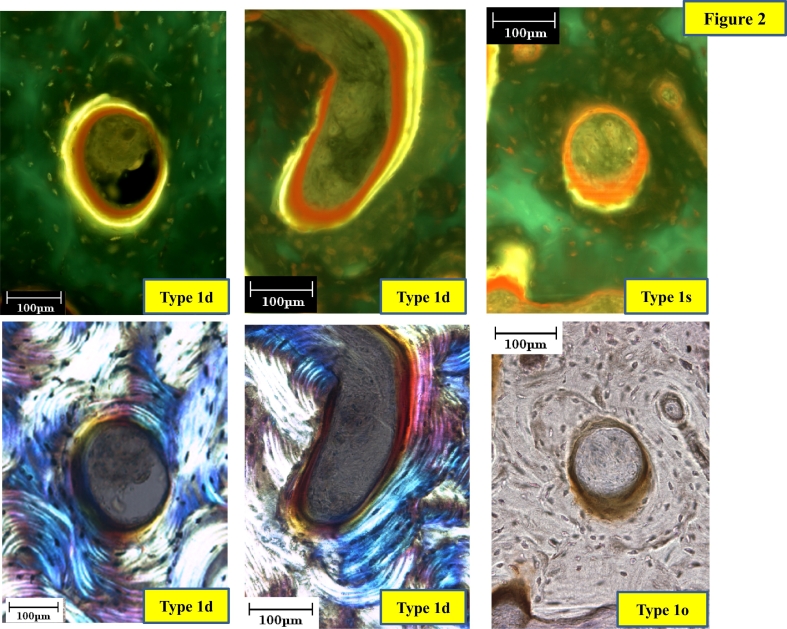
Type 1d, 1s and 1o pores. Type 1d pore is defined as the pore with double label in parallel lamellae to the inner surface, either circumferentially on the entire surface or partially with other phases of a remodeling cycle, which could be single label, osteoid, eroded and inert surface. Type 1s pore is also defined as the pore with single label in parallel lamellae to the inner surface, either circumferentially on the entire surface or partially with other phases of a remodeling cycle, which could be osteoid, eroded and inert surface, but not double label. Type 1d and 1s are to be observed under the fluorescent light. Type 1o with osteoid seam is observed under the bright field. In a case without labeling type 1d and 1s pores would be evaluated as type 1o under the bright light.

**Fig. 3:**
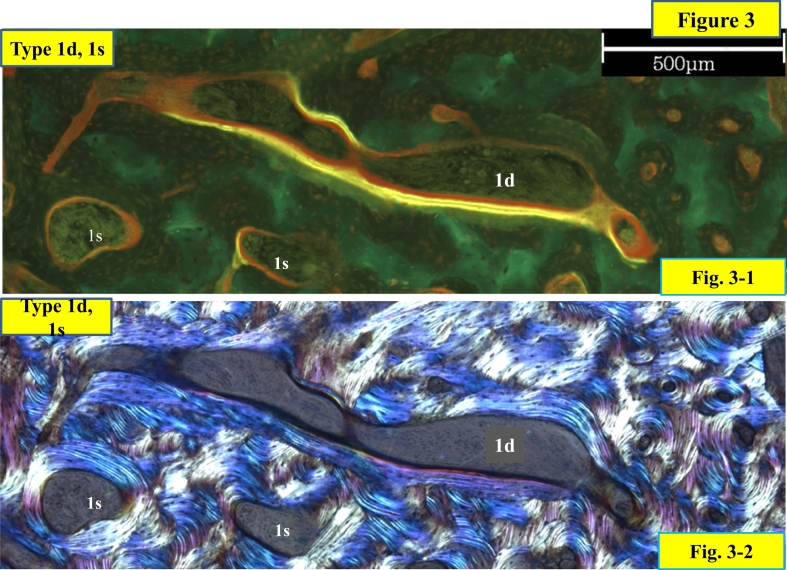
Transversely extended type 1 pore in the transverse section. Fig. 3-1 shows under the fluorescent light and Fig. 3-2 shows under the polarized light. Main feature of the 1d pore is double label surface, but also could show single label and osteoid surface. This type 1d pore shows a large pore with capability of lamellar bone formation. (Fig. 2 and this figure. A pore might show various phases of a remodeling cycle. Typing of the pore is summarized in Table 2, determined by observation under bright light, fluorescence and polarized light with an epifluorescent microscope (Olympus BX50) with a polarized attachment, mainly X100, occasionally X20, when the pore is too large to observe in a visual field under X100.).

**Fig. 4:**
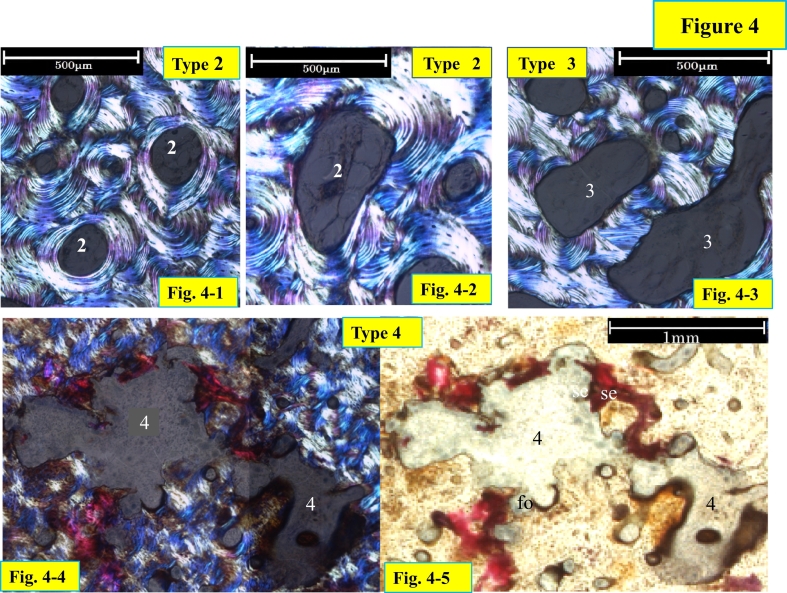
Type 2, 3 and 4 under polarized light microscopy. Type 2 pore shows parallel lamellae to the inner surface, either circumferentially on the entire surface (Fig. 4-1) or partially (Fig. 4-2) with other phases of a remodeling cycle, which could be osteoid, eroded, or inert surface. Nevertheless parallel lamellae are observed, bone formation has finished before labeling. Type 3 pore shows non-parallel lamellae to the inner surface, circumferentially on the entire surface. Type 4 pore shows irregular inner surface of the pore, which could be destroyed Haversian canal surface, and fragmented osteons (fo) structures, and contained bone debris and stained effusion (se).

**Fig. 5:**
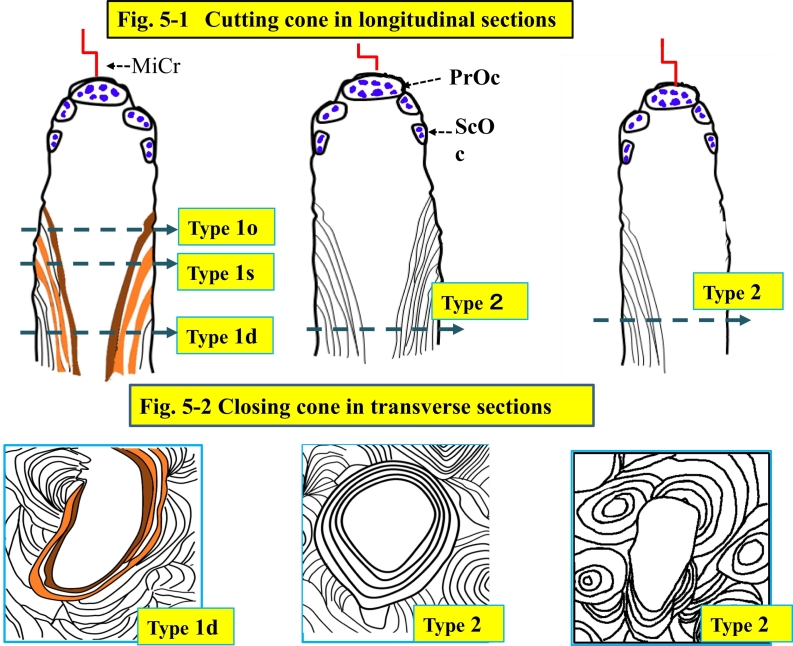

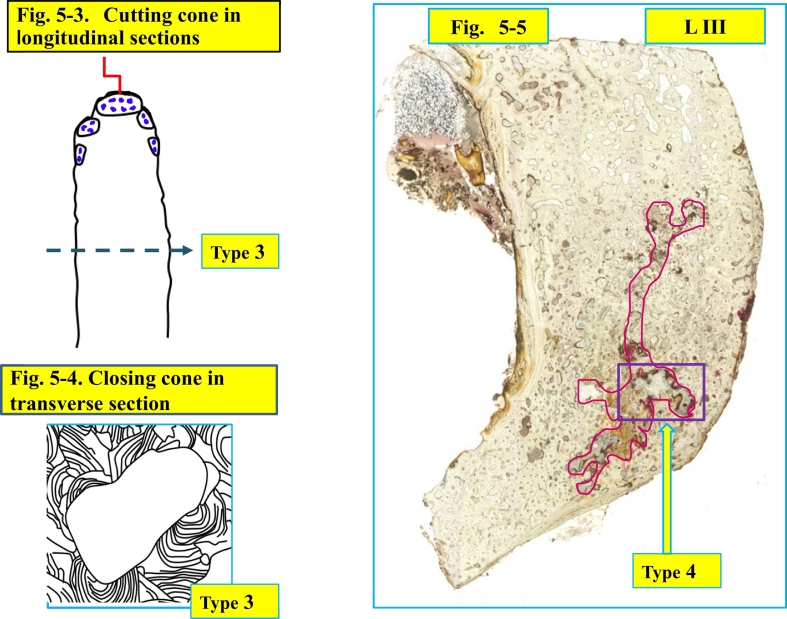
Illustration of the Haversian system that is the secondary osteon in the longitudinal section of the cortex of tubular bone. In osteonal remodeling there are five phases; activation, resorption, reversal/resorption, formation and quiescence. In atypical femoral fracture it has been documented accumulation of microcracks which activates resorption as targeted remodeling. When a few osteoclasts make a cutting cone, in which bone is resorbed in moving forward (upward in this figure) by primary osteoclasts which may advance through existing canals or cavities and in widening by secondary osteoclasts in the cutting cone. Fig. 5-1. The upper schemas show cutting cones in longitudinal section. PrOc: Primary Osteoclast, ScOc: Secondary Osteoclast, MiCr: Microcrack. Fig. 5-2. The lower schemas show the closing cones in transverse section. Label is only identified under the fluorescent light in undemineralized sections. Type 1: The cutting cone is followed by a closing cone with osteoid seam and labels. The number of labels or an osteoid seam without label depends on the level of the section. Type 1d pore is labeled twice both 1st and 2nd labelling, and the 1s pore is labeled only with the 1st label, when mineralization finishes before the 2nd label, since bone formation started after the first labeling. Type 1o indicates osteoid formation before 1st labeling in the closing cone. Type 2: The cutting cone is followed by a closing cone with lamellar bone formation, and the direction of lamellae is parallel to the inner surface of the pore, covered circumferentially its entire pore, or partially and the rest of the surface with non-parallel lamellae. When formation stopped before labeling, in transverse section, lamellar bone is observed without label or the osteoid seam. Fig. 5-3 Type 3: The cutting cone in the longitudinal section is followed by a resorption cavity and remains without forming bone in the closing cone. Fig. 5-4. In transverse section no lamellar bone parallel to the inner surface is observed. Fig. 5-5. Type 4 pore is formed with coalition of many large pores. The inner surface is composed with destructed osteons, interstitial lamellae, containing bone debris and effusion stained with a bone stain. There are many type 4 pores and large type 1 and 2 pores in the area surrounded red line. Type 4 pore in the purple box is shown in Fig. 4-4 under the polarized light and Fig. 4-5 under the bright light. (For interpretation of the references to colour in this figure legend, the reader is referred to the web version of this article.)

The patient was an 83 year-old Japanese woman with osteoporosis, treated with Risedronate and Alfacalcidol for the previous five years. She started to have left lateral thigh pain and claudication following a low-energy fall 4 months prior to admission. Radiographs revealed thickening of the lateral cortex and a transverse radiolucent fracture line in the diaphysis (Figs. 1-1, 4). In 2007 she had bilateral knee arthroplasty. In addition, the contralateral thigh showed significant femoral bowing. The fracture fulfilled the ASBMR Task Force revised criteria for atypical femoral fractures (Shane et al., 2010, Shane et al., 2014). By MRI, there was a high intensity signal on a T2WI image 1 cm long on the lateral cortex (Figs. 1-2, 3). Bone mineral density (BMD) of the left femoral neck and lumbar spine were 0.419 g/cm^2^ (% Young Adult Mean [YAM]; 53%) and 0.851 g/cm^2^ (%YAM; 84%) respectively. The patient had normal levels of bone resorption and formation markers except for low 25(OH) Vitamin D (Table 1). After admission, Risedronate was discontinued and Tetracycline hydrochloride was administered orally on a 02-08-02-08 schedule. As anterograde intramedullary nailing was difficult because of excessive femoral bowing, a corrective osteotomy and intramedullary nailing were performed. During surgery, the site of the transverse radiolucent fracture line was soft and fragile, and transparent effusion was observed by finger-pressure to the protruded cortex. After reaming, a biplanar closed wedge osteotomy (longitudinal width: 5 mm/angle: 10 degrees) and osteosynthesis were performed using an anterograde femoral nail. In order to bridge the gap of the osteotomy site and to acquire rotational stability, decortication and local site plating fixation were added (Fig. 1-5). The bone fragment excised at osteotomy broke into two pieces on removal and was preserved for bone histomorphometry.

**Table 1:** Biochemical markers on admission.

Parameters	Results	Reference range
Serum calcium (mg/dL)	10.2	8.7–10.3
Serum phosphorous (mg/dL)	3.5	2.5–4.7
Serum alkaline phosphatase (U/L)	219	115–369
Serum TRACP-5b (mU/dL)	335	120–420
Serum P1NP (lg/L)	17.9	17.0–64.7
Serum ucOC (ng/mL)	2.48	<4.50
Serum homocysteine (nmol/mL)	10.8	5.1–11.7
Serum pentosidine (pg/mL)	0.15	9.2–43.1
25(OH)Vit.D(ng/ml)	17.0	30–100

P1NP = procollagen type 1N-terminal propeptide.

TRACP-5b = tartrate-resistant acid phosphatase type 5b.

ucOC = uncarboxylated osteocalcin.

**Table 2:** Classification of intracortical pores.

Type	Label^a^/osteoid^b^	Characteristics of inner surface of pores	
		Direction of lamellae to the inner surface^c^	LmB·S: circumferential formation or partial formation, possibly together with
1d	**Double label**	**Parallel**	sLS, ES, OS
1s	**Single label**	**Parallel**	ES. OS
1o	**Osteoid**	**Parallel**	ES
2	**Non**	**Parallel**	ES, IS
3	**Non**	**Non parallel**	IS
4	**Non**	**Irregular**	Labeled fragment, diffuse labeling

A term in bold letters is essential conditions for the type.

sLS: single label surface ES: eroded surface OS: osteoid surface IS: inert surface.

a Fluorescent light.

b Bright light.

c Plarrised light.

### 2.2 Bone histomorphometry

#### 2.2.1 Cortical bone assessments

##### 2.2.1.1 Sectioning and staining

The lateral osteotomy piece, was fixed in 70% ethanol, stained with Villanueva bone stain and embedded in methyl methacrylate without decalcification (Villanueva and Lundin, 1989).The embedded lateral piece was sectioned at a thickness of 300 μm in the horizontal direction perpendicular to the shaft using Maruto's Micro Cutter MC 201. The bone sample was ground to a thickness of 30 μm (Section L I). After section L I was made, a radiograph of the rest of the block showed a linear radiolucent zone at the edge of the block, revealing an incomplete fracture line of the lateral cortex. Two more sections were made (Sections L II and L III) based on the finding of the fracture line in this region.

##### 2.2.1.2 Observation, criteria and assessment

In this case, intracortical pores in the lateral cortex have demonstrated an abnormal remodeling at a glance of observation that was many enlarged pores. Subsequently, a novel criteria has developed in focusing simply to assess the size of each pore how much cumulative bone resorption had occurred and whether bone formation is currently being occurred, shown with label, had occurred before labeling period, but have stopped formation and inert at the surgery, or had not occurred after reversal-resorptive period. Since one would image “a quiescent surface” may be covered with osteoblastic linage cells in normal remodeling cycle, a term, “inert” was used in the report, showing neither resorption nor formation.

Under bright field at X 20 magnification, the size of each pore was measured by a histomorphometric assessment set, “TP Measure” (System Supply, Nagano, Japan), using an epifluorescent microscope (Olympus BX50, Olympus Corporation, Tokyo, Japan) with a polarized attachment. Simultaneously, each pore type was classified under polarized light, at both X20 and X100 magnification. A map of each section under polarized light was made combining 150–250 visual fields under X100 magnification.

Type 1d pore is defined as the pore with double label (dLS) in parallel lamellae to the inner surface, either circumferentially on the entire surface or partially with other phases of a remodeling cycle, which could be single label (sLS), osteoid (OS), eroded (ES) and inert surface (IS). Type 1s pore is also defined as the pore with single label (sLS) in parallel lamellae to the inner surface, either circumferentially on the entire surface or partially with other phases of a remodeling cycle, which could be osteoid, eroded and inert surface, but not double label surface. Type 1o has an osteoid seam, observed under bright light field, supplemented assessment of type1d or 1s, but not counted separately because of the thickness of the sections.

Type 2 pore is defined as the pore with parallel lamellae to the inner surface, either circumferentially on the entire surface or partially parallel, but with non-parallel lamellae on the rest of the surface. Type 3 pore shows non-parallel lamellae on its entire inner surface.

Type 4 pore is a cavity with the irregular and disrupted surface composed of fragmented osteonal structure. This may be results of coalition of many large pores, containing fragments of lamellar bone and debris inside (Figs. 4-4, -5, 5-5, 10-1). Each pore was assessed its type and measured its size.

Fig. 5-1 and -3 were illustrated in longitudinal section a cutting cone with primary osteoclasts at the tip targeting a microcrack and secondary osteoclasts at the side wall and pore with double label (type 1d), single label (type 1s) and osteoid seam (1o), respectively, and with lamellae parallel to the inner surface of the pore (type 2) or without parallel lamellae (type 3). Fig. 5-2 and -4 illustrate, in the transverse section of a closing cone, the same relation between types 1, 2 and 3 in the transverse plane of the closing cone.

### 2.3 Finite element analysis (FEA)

Patient specific finite element models were created using preoperative CT data (0.625 mm slice) of the affected femur. Stress concentrations in the femoral shaft were analyzed using “MECHANICAL FINDER” software (Research Center of Computational Mechanics, Inc., Tokyo, Japan). The mesh size was 2 mm and we used Keyak's model to define the material constants (Keyak et al., 1998). Poisson's ratio for each element was set as 0.4. The line from the center of the femoral head to the center of the distal condyle of the femur was considered the loading axis. The distal condyle was constrained, and a load equal to the patient's weight was applied to the center of the femoral head. According to the method of Oh et al., we analyzed the distribution of tensile stresses, and maximum and mean values of maximum principal stress (MPS) by data extraction from a 70 mm section in the middle of the femoral diaphysis (Oh et al., 2014; Oh et al., 2017; Tano et al., 2019), we analyzed the distribution of tensile stresses for the medial and lateral cortex of the femur, and compared this to bone histomorphometric findings.

### 2.4 Statistical analysis

A Chi-square test after a Ryan's multiple comparison test was performed for the number of the pores in the three sections to determine differences among 1d, 1s, 2 and 3 pores. * *p* < 0.05 and ** *p* < 0.01 were considered statistically significant.

Non-parametric one way ANOVA, Kruskal-Wallis test, and post hoc tests, Mann-Whitney *U* tests with Bonferroni correction was used for the comparison of pore size among type 1d, 1s, 2 and 3. And for the comparison of pore size per mm^2^ of the lateral cortex among pore type 1d, 1s, 2 and 3 between tensile and compressive area. * p < 0.05 and ** p < 0.01 were considered statistically significant. R version 3.6.2(The R Foundation for Statistical Computing, Austria)was used for all statistical analyses.

## 3 Results

### 3.1 Patient clinical outcome

Two weeks after surgery, low-intensity pulsed ultrasonography (LIPUS) (Lou et al., 2017) and daily Teriparatide (20 μg/day) (Kendle et al., 2018) therapy were started to promote bone healing. The patient was permitted partial weight bearing two weeks post-operatively, and full weight bearing at six weeks. Three months post-operatively the plate was removed to prevent plate inhibition of bone healing. At 10 months, the osteotomy site showed the process of union, but the patient developed an acute subarachnoid hemorrhage and died a week later.

### 3.2 Bone histomorphometry (Dempster et al., 2013)

#### 3.2.1 Specimen of the lateral cortex and characteristics of three sections

##### 3.2.1.1 Observation of three sections

In this study three ground sections are available for observation and measurements, and each section may have some characteristics because of its location to the radiolucent line (the incomplete fracture line) in the lateral cortex. The L I section locates about 1 mm apart from the L II section which include the edge of the radiolucent line and the L III section include the other side of the edge. The radiolucent line is about 1 mm in thickness, locates between L II and L III, lost in preparation of the sections because of a rotary saw, 600 μm in thickness. At a glance of these sections there are many and large pores are observable in the L I section (Fig. 8-3) and further large cavity-like pore in L II and L III (Fig. 10-1). Then, a novel criteria was developed to know the characteristics of pores, then measured.

Measurements of pores and histomophometric results and discussion.

**Fig. 6:**
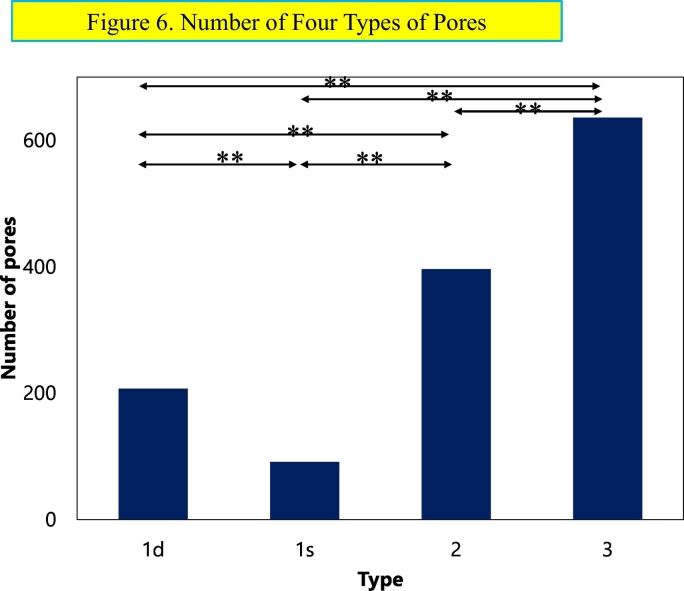
Number of four types of pores. Comparisons of the number of four types of the pores, among type 1d, 1s, 2 and 3 pore in all three sections. Number of the type 1s pore is the lowest, 1d pore is next, followed by the type 2 pore, and the type 3 pore is the highest. Each type pore is statistically different from others.

A number of four types of pores is shown in Fig. 6. The number of the type 1s pore is the lowest and next the type 1d pore and the type 2 pore, and the type 3 pore is the highest among four types.

The numbers of these four types are different statistically each other.

**Fig. 7:**
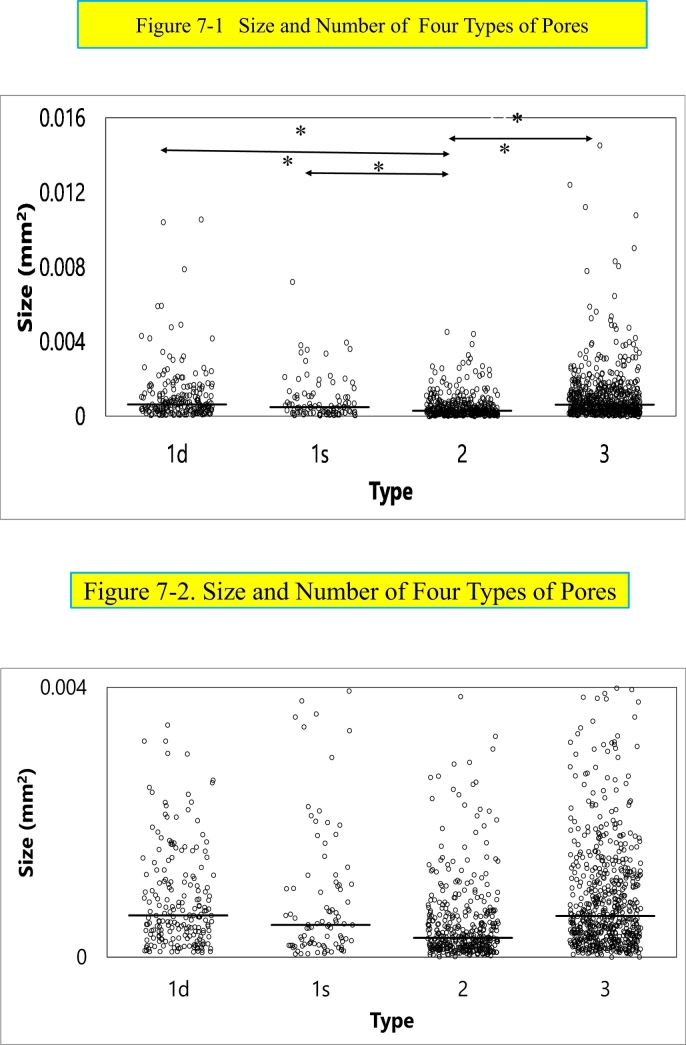
Size and number of four types of pores. Size and number of four types of pores are shown in the plot-dot figures (Fig. 7-1 in the high scale and 7-2 in the low scale) showing the median values. The type 2 pore is the lowest and the type 3 pore is the highest median value. *(Non-parametric one way ANOVA, Kruskal-Wallis test, and* post hoc tests, Mann-Whitney U tests with Bonferroni correction was used for the comparison of pore size and number. * p < 0.05 and ** p *<* *0.01 were considered statistically significant.)*

Size and number of four types of pores are shown in the plot-dot figures (Fig. 7-1 in the high scale and 7-2 in the low scale) showing the median values. The type 2 pore is the lowest and the type 3 pore is the highest median value. Porosity (TtPo·Ar/B·Ar) of the three sections (L I, L II and L III/3) is 31.4%.

#### 3.2.2 Enchondral bone formation

**Fig. 8:**
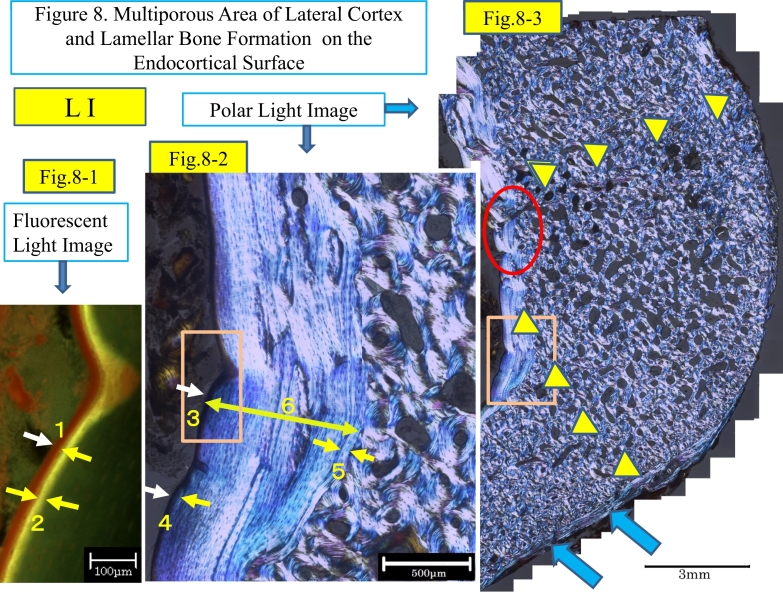

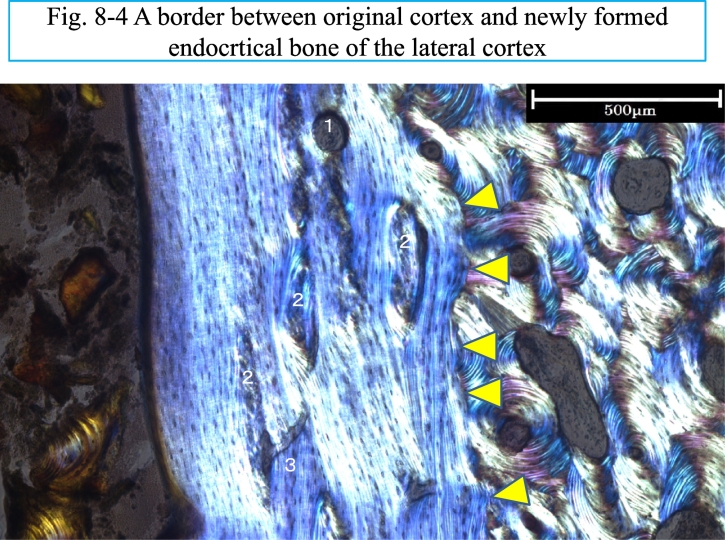
Three images of L I section, about 1 mm apart from the radiolucent line are shown from the left, in higher, middle and to the right in low magnification. Fig. 8-1. A fluorescent light image shows endocortical surface of the lateral cortex, bordered with the marrow cavity. At the most superficially osteoid seam, deep red in color, is indicated by the numeral 1, pinched by two short white and yellow arrows. The next layer is a label, shown as single yellow label, indicating the numeral 2, pinched by a pair of short white and yellow arrows. Although the label looks like single in print, it was microscopically observed as double because of the thickness of the ground section (35 μm) and the inter-label distance was measured. Then, the endocortical mineral apposition rate (EcMAR) was calculated. Fig. 8-2. An enlarged image of the endocortical surface of the lateral cortex under polarized light shows endocortical lamellar bone to the left and compact bone with enlarged pores to the right. The numeral 3 indicates the osteoid surface by the short white arrow. The numeral 4 indicates the osteoid seam, shown by a pair of short white and yellow arrows. The numeral 5 indicates the first layer of lamella, bordered with the compact bone. The numeral 6, a double headed yellow bar, indicates maximum endocortical lamellar bone thickness (EcLmB·Th). This is the distance from the numeral 3, the osteoid seam surface, indicated by a short white arrow to the first lamellar layer, indicated by the numeral 5. EcLmB·Th is 848 μm and EcMAR is 0.85 μm/day. Then, formation period, (EcLmB·Th)/(EcMAR), is 998 days. The first layer numbered 5 was formed about 2.7 years prior to the osteotomy and the osteoid seam numbered 4 (same as the number 1 in Fig. 8-1) is being formed at the time of the corrective osteotomy. The square-box of the endocortical surface in Fig. 8-2 is shown under fluorescent light in Fig. 8-1. The formation period might be still underestimated since growth arrest might have occurred in formation of endosteal lamellar bone. Fig. 8-3. This is a collective image of about 200 visual fields at X100 magnification under polarized light. There are many large pores in the triangular area of the lateral cortex, surrounding the point of interrupted lamellar bone, indicated by a red oval on the endocortical surface, and to the area indicated by two series of small yellow triangle marks. All pores are classified as to type and size (Fig. 7-1, and -2). On the periosteal surface at the lower part of the cortex, the remnants of lamellar bone are indicated by the blue arrows. The area designated in the square box of the endocortical surface is shown at higher magnification in Fig. 8-2. Fig. 8-4. Fig. 8-4 shows a border between the original lateral cortex and the newly formed endocortical bone. In the figure there are five protuberances (indicated with yellow triangles) of lamellar bones into partially resorbed osteons in the lateral cortex, as if there were Howship's lacunae prior to formation of lamellar bone. The numeral 1 in the lamellar bone is a round resorption cavity. The numeral 2 is an interlamellar breakage and 3 shows lamellar interruption. (For interpretation of the references to colour in this figure legend, the reader is referred to the web version of this article.)

On the endocortical surface of L I (Fig. 8), a zone of lamellar bone (EcLm.B) was observed. The maximum thickness of the zone was 848.26 μm and its mineral apposition rate was 0.85 μm/day. The formation period of the zone was about 1000 days. There is interruption of the lamellae at the middle of the endocortical surface, indicated with a red oval line. On the periosteal surface of L I, lamellar bone is not observed microscopically except for a remnant (Fig. 8-3), although it is recognized on the x-ray (Fig. 1-4). This was probably removed at surgery to make the femoral surface flat for plate fixation. Fig. 8-4 shows a border between the original lateral cortex and the newly formed endocortical bone. In the figure there are five protuberances (indicated with yellow triangles) of lamellar bones into partially resorbed osteons in the lateral cortex, as if there were Howship's lacunae prior to formation of lamellar bone.

In an initial half of the endosteal bone formation from the layer of protuberances as a base of lamellar bone there are a few irregular lamellae, probably interlamellar breakage are observed, not like scalloping cement lines or growth arrest lines. About one third of the lamellar bone seems to have been formed continuously on the wider area of the endosteal surface.

### 3.3 Finite element analysis (FEA)

**Fig. 9:**
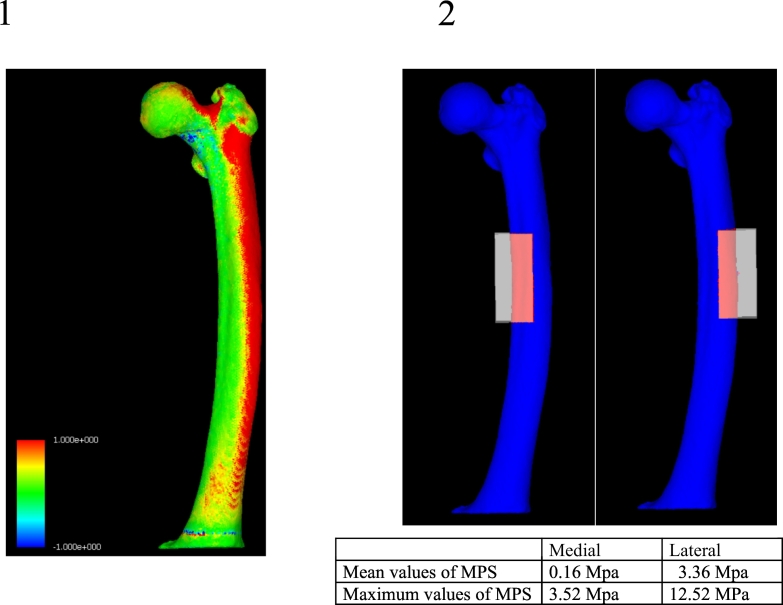
FEA image of the left femur shown in AP view. Tensile force is shown in red. Maximum principal stress (MPS) on the lateral cortex was higher than that on the medial cortex.

FEA color charts showed a marked tensile stress concentration on the lateral surface throughout the length of the femoral shaft. The mean values of MPS were 0.16 MPa on the medial cortex of the femoral shaft and 3.36 MPa on the lateral cortex. Maximum values of the MPS were 3.52 MPa on the medial cortex and 12.52 MPa on the lateral cortex (Fig. 9).

Therefore, comparing between medial cortex and lateral cortex, significantly large tensile stress was applied to the lateral cortex.

### 3.4 The relationship of FEA, CT image and histological section

Fig. 8 shows a transverse image of FEA, CT and histological section (L II).

**Fig. 10:**
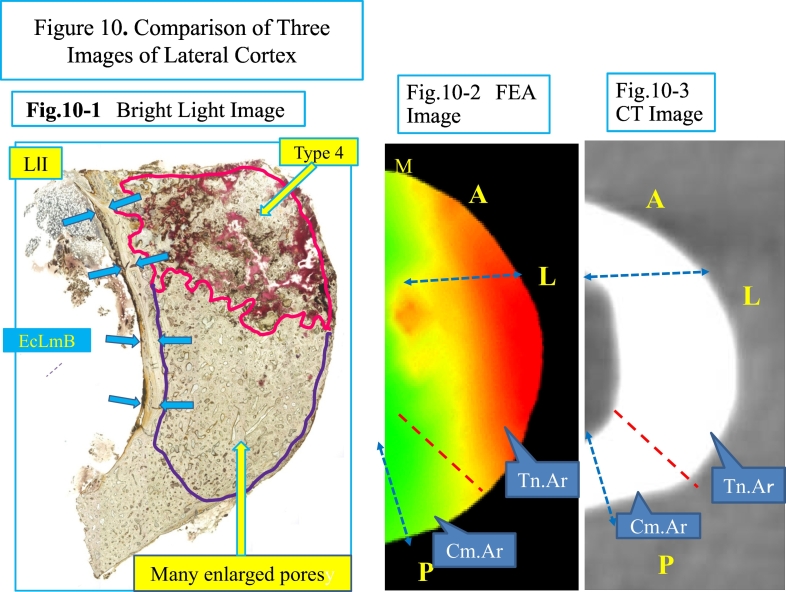
Comparison of three images. Fig. 10-1. A bright light image of section L II (transverse direction) just next to a radiolucent line (partially included) on X-ray with periosteal and endocortical hypertrophy. No periosteal bone formation is observed histologically. In the upper one quarter of the section is a type 4 pore, which contains fragmented lamellar bone and debris. The middle half of the cortex contains many enlarged pores of types 1, 2 and 3. The lower quarter of the lateral cortex contains smaller sized pores. Endocortical Lamellar Bone (EcLmB) is indicated by four pairs of blue arrows. Fig. 10-2. On the FEA image, about three-quarters is under tensile force (indicated in red), which matches the undemineralized bone section with numerous pores. The anterior part of the cortex is not included in the section as it was left on the edge of the shaft to create stability of the fixation from the osteotomy and intramedullary nailing. Fig. 10-3. A CT image of the midshaft of the femur. Fig. 10-2, -3 are images in transverse direction, corresponding the area of the histological section with two dotted lines. A: anterior; P: posterior, and L: lateral. In Fig. 10-2 the red area is showing tensile area (Tn.Ar) whereas the light green area is the compressive area (Cm·Ar). (For interpretation of the references to colour in this figure legend, the reader is referred to the web version of this article.)

**Fig. 11:**
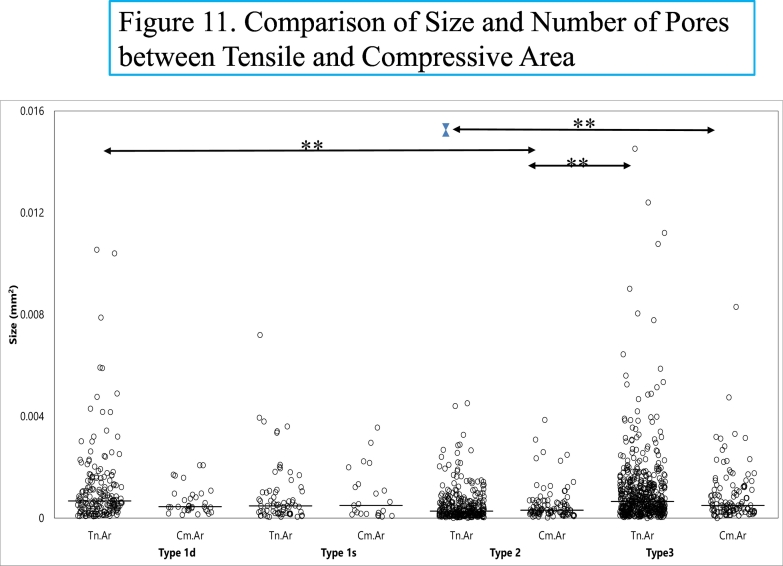
Comparison of size and number of four types of Pores between tensile and compressive area in the lateral cortex. Non-parametric one way ANOVA, Kruskal-Wallis test, and post hoc tests, Mann-Whitney *U* tests with Bonferroni correction was used for the comparison of pore size per mm^2^ of the lateral cortex among pore type 1d, 1s, 2 and 3 between the tensile and the compressive area. Type 1d, Tn.Ar is statistically larger than type 2, Cm·Ar; Type 2, Tn.Ar is higher than type 3, Cm. Ar, and type 3, Tn. Ar is higher than type 2, Cm.Ar. (* *p* < 0.05 and ** *p* < 0.01 were considered statistically significant.)

Many large pores were observed histologically under bright light (Fig. 8-3). Type 4 pores and enlarged pores, type 1, 2 and 3, were mostly found in the upper three quarters of the section (Fig, 5-5, 8-3, 10-1). The extent of the area with enlarged pores matches a marked tensile stress concentration area on the lateral cortex based on the FEA image, shown in red (Fig. 10-2), and an area of periosteal and endocortical hypertrophy on the transverse image of the CT (Fig. 10-3). MRI shows a high intensity area, 1 cm in length (Fig. 1-3) in the lateral cortex. On an image of histological section a line was drawn between a quarter point of periosteal surface and of endosteal surface (Fig. 10-2, -3). The line divides red area (tensile area, Tn.Ar) and light green area (compressive area, Cm·Ar). Each type of pores was compared between Tn.Ar and Cm·Ar per mm^2^ of the lateral cortex. The size and the number of each pore per mm^2^ are larger in the tensile area than the compressive area (Fig. 11).

## 4 Discussion

In this case, there was a history of total knee arthroplasty (TKA) 10 years previously, but the diagnosis of an incomplete atypical femoral fracture was made based on ASBMR criteria following a minor injury. Due to severe lateral bowing, a corrective osteotomy with a triangular wedge for stability and intramedullary nailing were performed. The wedge removed during surgery was assessed by histomorphometry.

Most importantly in this study, we were able to compare an image of the lateral cortex by the finite element method with that by bone histomorphometry after tetracycline bone labeling. There are only a few reports on bone histomorphometric findings at the fracture site in AFF. Jamal et al. (2011) reported findings after tetracycline double labeling in a specimen obtained just below the point of fracture, which confirmed bone formation. Schilcher et al. (2011) reported histology of eight cases of AFF, which revealed channels along the fracture line in longitudinal sections. These findings are consistent with what we have found in the lateral cortex of the femur.

At the beginning of osteonal remodeling in cortical bone, microcracks produced by mechanical loading are targeted by osteoclasts for resorption (Frost, 1960; Burr et al., 1997; Mashiba et al., 2001; Parfitt, 2002).

These osteoclasts in the tip of the cutting cone, called primary osteoclasts, take the role of longitudinal advancement at a rate of about 40 to 50 μm/day (Norimatsu, 1971; Jaworski and Lok, 1972; Takahashi and Norimatsu, 1976).

### 4.1 Presence of reversal-resorptive phase

After longitudinal extension of the cutting cone in the initial resorption phase, Delaisse's group (Delaisse, 2014; Andersen et al., 2013; Andreasen et al., 2015; Abdelgawad et al., 2016)proposed a reversal-resorptive (RvRs) phase in which reversal cells and osteoclasts appeared on the wall of the tunnel portion of the cutting cone. These secondary osteoclasts play a role in widening the cutting cone. Based on cutting cones obtained from femurs of 9 patients and fibulae of 10 patients, Lassen et al. (2017) showed that bone matrix was subjected to several resorption episodes, separated by reversal periods during which increasing numbers of reversal/osteoprogenitor cells were recruited. Once a threshold reversal/osteoprogenitor cell density is reached, bone formation is initiated and resorption is switched off. They proposed a model in which the rate of reversal/osteoprogenitor cell recruitment slows the extent of resorption.

### 4.2 Widespread variation of reversal/resorptive phase

In this case the amount of cortical bone, resorbed in osteonal remodeling in the lateral cortex through both initial and secondary resorption is not clear, but secondary osteoclasts may play a major role in increasing pore size. Lassen et al. (2017) also reported widespread variation in the absolute length of the RvRs zone among measured BMUs, indicating great variations in the time lag for starting bone formation after the period of initial resorption (fivefold variation between the smallest and the largest RvRs length). The longer the RvRs surface, the larger the diameter of the cutting cone. Norimatsu (1971) examined 298 cutting cones of long tubular bones of fore- and hindlimbs and ribs of mongrel dogs, and reported high correlation (*r* = 0.48–0.90, *p* < 0.001–0.002) between a linear rate of longitudinal bone resorption and osteon diameter (Norimatsu, 1971; Takahashi and Norimatsu, 1976). This relation reflects that the longer the exposure of the RvRs surface to osteoclasts, the more bone is removed. The osteoblast density at the onset of bone formation is independent of the length of the RvRs of different cutting cones (Lassen et al., 2017). In this case there is also a large variation of pore size in the lateral cortex.

### 4.3 Successive bone formative phase

**Bisphosphonates** strongly inhibit bone resorption, but also strongly decrease bone formation. The deceased formation is commonly thought to be due to the mechanism maintaining the resorption/formation balance during a remodeling cycle. Jensen et al. (2021) has reported an evidence for an additional mechanism where bisphosphonates actually impair the onset of bone formation after resorption, based on morphometric assessment on the activities reversing resorption to formation. They have compared a bisphosphonate increases the prevalence of eroded surfaces characterized by reversal cells/osteoprogenitors at low cell density and remove from active bone surfaces, although cell expansion on eroded surfaces is indispensable to start formation. Furthermore, alendronate decreases the coverage of these eroded surfaces by remodeling compartment canopies, a putative source of reversal cells/osteoprogenitors.

Although pore size is increased, in this case, the pore can still form lamellar bone with double or single label (type 1d and 1s pores), observed in the periphery of the lateral cortex of the L I section. The small size pores show the largest number of type 1, and larger size pore still shows type 1 (Fig. 7-1, -2). Large sized 1d and 1s type pores are 6 to 7 fold larger than the median size still maintain the capability for bone formation, suggesting that “once a threshold reversal/osteoprogenitor cell density is reached, bone formation is initiated and resorption is switched off”(Lassen et al., 2017. The pores with lamellar bone covering the inner surface of the pore without label may suggest cessation of bone formation prior to labeling (type 2 pore). Type 2 pores not only have parallel lamellae with bone formation on the entire inner surface, but some pores show formation only on a part of the inner surface; the rest of the inner surface could be inert (Fig. 4-2), This probably means that the reversal/osteoprogenitor cell density locally is different within the pore. Pores without lamellar bone formation on their inner surface were named type 3 pores. When secondary osteoclasts have a long lifespan or are successively recruited, the widening may continue over a prolonged period. When secondary osteoclasts are not recruited, widening stops, and no successive bone formation occurs (type 3, Fig. 4-3).

When bone formation is switched on, the osteon shows radial closure and forms the Haversian canal. The MAR of type 1d pores is normal, but bone formation does not persist to create a normal sized Haversian canal. Based on the cross-sectional area of rib osteons from age 20 to 70 years of age (Landeros and Frost, 1964), and femoral osteon size of an 80 year-old female (Yamamoto et al., 2018), the cross-sectional area of the Haversian canal is about 10% of the cross-sectional area of the secondary osteon. Pore size is much larger than normal canal size, probably due to incomplete coupling and poor osteoprogenitor cell density.

### 4.4 Development of pores and their coalition

Bisphosphonates used to treat osteoporosis decrease osteoclast function, resulting in accumulation of microcracks (Mashiba et al., 2001), which might cause weakness of the femoral shaft resulting in atypical femoral fracture (Shane et al., 2014). In this case targeted remodeling of microcracks developed into large pores with widespread variation. Although lamellar bone was still forming at the time of biopsy in type 1 pores, insufficient bone was formed in type 2 pores and no bone formation occurred in type 3 pores. This may suggest incomplete or no coupling had occurred just after the reversal/resorption phase. A radiolucent fracture line between the L II and L III sections revealed a region where coalescence of pores had occurred, resulting in large type 4 pores containing fragments of destroyed lamellar structure and debris.

### 4.5 Periosteal and endocortical lamellar bone formation

**Fig. 12:**
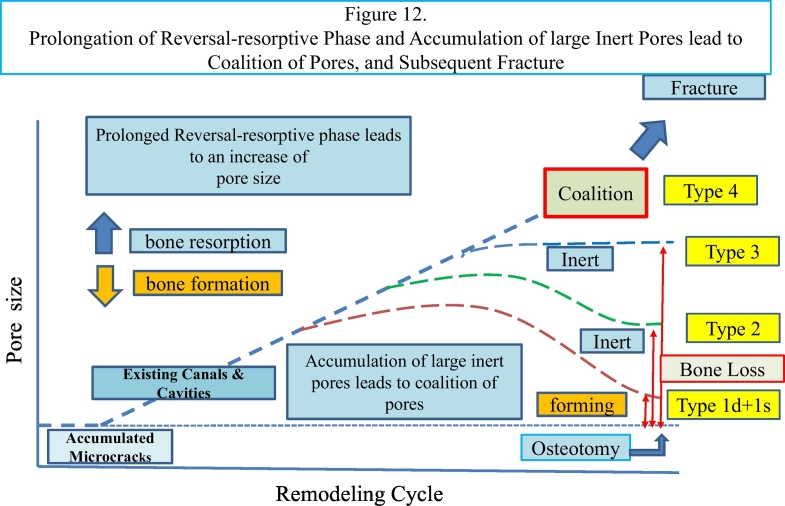
Prolongation of reversal-resorptive phase and accumulation of large inert pores leads to coalition of pores, and subsequent fracture. Accumulated microcracks might have initiated bone resorption as targeted remodeling and propagated the process to existing canals and cavities. Focal biomechanical environment in the lateral cortex might lead to prolongation of reversal-resorptive phase, resulting in an increase of pore size. Subsequently, BMUs may change to start bone formation, and continued to form lamellar bone until the period of labeling.(type 1 pore). Some BMUs might have switched from bone resorption to bone formation of lamellar bone, but stopped in the formative phase before labeling. Thereafter, the inner surface of the pore would be inert (type 2 pore). Other BMUs had started to resorb bone, and increased its size in resorptive phase, but did not switch to bone formation. Then the inner surface of the pore would be inert (type 3 pore). Many large sized inert pores may lead to coalition, increased its size (type 4), resulting in subsequent fracture.

In osteonal remodeling lamellar bone is formed. Intracortical bone loss near and at the fracture line of the lateral cortex, where type 4 pores accumulated in a narrow portion of the shaft, may have weakened the bone, resulting in compensatory periosteal and endocortical lamellar bone formation that began three years prior to osteotomy, although this may be still underestimated. On both periosteal and endocortical surfaces thickening of bone was radiologically observed in the transverse radiolucent linear zone. A border between the original lateral cortex and the newly formed endosteal lamellar bone may be a type of the “cement line”, but it is primarily a term for formation of secondary osteon (Skedros et al., 2005; Okada et al., 2013). It seems, in this case, endosteal lamellar bone formation is modeling-based formation, started with resorption of the lateral cortex, but not remodeling-based formation (Fig. 12).

In recent years there have been several reports using FEA to characterize stresses around an AFF (Sasaki et al., 2012; Maratt et al., 2014; Oh et al., 2020; Demirtas et al., 2020). Oh et al. demonstrated that significant tensile stress caused by bowing of the femoral shaft is associated with AFFs. They further biopsied the lateral cortical bone (about 1 × 1 cm) of the AFF site, performed the bone histomorphometry, have assessed the local bio-activity. Our study was similar, but our method allowed cortical bone collection to be performed approximately all around and bone labeling with tetracycline, which was more detailed evaluation. We were also able to demonstrate that the tensile stress of the femoral shaft and that the maximum principal stress differ significantly between the medial and lateral cortices. Furthermore, when the bone cross-section was compared to the CT axial slice and the axial image from the FEA, type 4 pores had accumulated at the tensile stress concentration site on the lateral femoral cortex (Fig. 10).

This study had some limitations. First, this was a study of only one case. We sometimes encounter AFF and perform surgery, but it is common to treat AFFs with intramedullary nailing alone, and it is not easy to collect nearly circumferential cortical bone from the fracture site. Second, the clinical course of this fracture is unknown due to death of the patient. Third, the FEA load conditions only apply to the stance phase, and stresses were not evaluated during the swing phase.

## 5 Summary

The presence of large pores in regions of developing AFFs may mean prolonged activity of secondary osteoclasts, together with loss of complete or incomplete coupling with bone formation. With the gradual accumulation of large pores and their coalition, in combination with high tensile stresses on the lateral femoral cortex, AFF eventually developed.

## Ethics

This study design was approved by the ethics review board of the first author's institution (H26-096). The informed consent was obtained from the patient, complying with the principles laid down in the Declaration of Helsinki. The patient is completely anonymous, protecting the privacy and dignity.

## CRediT authorship contribution statement

**Fumitaka Hirano:** Conceptualization, Methodology, Software, Validation, Formal analysis, Investigation, Resources, Writing – original draft, Writing – review & editing. **Kayoko Furukawa Okuma:** Conceptualization, Methodology, Validation, Project administration. **Yukichi Zenke:** Supervision, Conceptualization, Resources. **Kunitaka Menuki:** Supervision, Conceptualization, Investigation. **Hideo Ohnishi:** Supervision, Conceptualization. **Fumio Fukuda:** Conceptualization, Software, Resources. **Akinori Sakai:** Supervision, Conceptualization. **Noriaki Yamamoto:** Supervision, Conceptualization. **Taketoshi Shimakura:** Methodology, Software, Validation, Data curation. **Hiroshige Sano:** Supervision, Conceptualization. **Yuta Tokunaga:** Software, Formal analysis. **Hideaki E. Takahashi:** Supervision, Conceptualization, Methodology, Validation, Writing – original draft, Writing – review & editing.
